# Postlicensure Safety Surveillance of Recombinant Zoster Vaccine (Shingrix) — United States, October 2017–June 2018

**DOI:** 10.15585/mmwr.mm6804a4

**Published:** 2019-02-01

**Authors:** Elisabeth M. Hesse, Tom T. Shimabukuro, John R. Su, Beth F. Hibbs, Kathleen L. Dooling, Ravi Goud, Paige Lewis, Carmen S. Ng, Maria V. Cano

**Affiliations:** ^1^Epidemic Intelligence Service, CDC; ^2^Division of Healthcare Quality Promotion, National Center for Emerging and Zoonotic Infectious Diseases, CDC; ^3^Division of Viral Diseases, National Center for Immunization and Respiratory Diseases, CDC; ^4^Division of Epidemiology, Center for Biologics Evaluation and Research, Food and Drug Administration, Silver Spring, Maryland.

Recombinant zoster vaccine (RZV; Shingrix), an adjuvanted glycoprotein vaccine, was licensed by the Food and Drug Administration (FDA) and recommended by the Advisory Committee on Immunization Practices for adults aged ≥50 years in October 2017 (*1*). The previously licensed live-attenuated zoster vaccine (ZVL; Zostavax) is recommended for adults aged ≥60 years. RZV is administered intramuscularly as a 2-dose series, with an interval of 2–6 months between doses. In prelicensure clinical trials, 85% of 6,773 vaccinated study participants reported local or systemic reactions after receiving RZV, with approximately 17% experiencing a grade 3 reaction (erythema or induration >3.5 inches or systemic symptoms that interfere with normal activity). However, rates of serious adverse events (i.e., hospitalization, prolongation of existing hospitalization, life-threatening illness, permanent disability, congenital anomaly or birth defect, or death) were similar in the RZV and placebo groups (*2*). After licensure, CDC and FDA began safety monitoring of RZV in the Vaccine Adverse Event Reporting System (VAERS) (*3*). During the first 8 months of use, when approximately 3.2 million RZV doses were distributed (GlaxoSmithKline, personal communication, 2018), VAERS received a total of 4,381 reports of adverse events, 130 (3.0%) of which were classified as serious. Commonly reported signs and symptoms included pyrexia (fever) (1,034; 23.6%), injection site pain (985; 22.5%), and injection site erythema (880; 20.1%). No unexpected patterns were detected in reports of adverse events or serious adverse events. Findings from early monitoring of RZV are consistent with the safety profile observed in prelicensure clinical trials.

VAERS is a national passive surveillance system for adverse events after administration of U.S.-licensed vaccines and is coadministered by CDC and FDA (*3*). VAERS accepts reports from health care providers, vaccine manufacturers, and the public. Signs and symptoms of each adverse event are coded using Medical Dictionary for Regulatory Activities (MedDRA) terminology (*3*). A single VAERS report might be assigned more than one MedDRA Preferred Term*; these terms are not necessarily medically confirmed diagnoses. VAERS reports are classified as “serious” according to Code of Federal Regulations Title 21 Section 600.80.^†^ Medical records are requested for reports of serious adverse events, including autopsy findings and death certificates for reported deaths.

CDC and FDA investigators conducted descriptive analyses of reports to VAERS involving RZV for the period October 20, 2017–June 30, 2018. Physicians reviewed reports (as well as medical records and other documentation when available) for 22 prespecified outcomes, which included conditions of general interest for vaccine safety and conditions identified as possible or theoretical safety concerns from prelicensure clinical trials (Supplementary Table 1, https://stacks.cdc.gov/view/cdc/62214) (Supplementary Table 2, https://stacks.cdc.gov/view/cdc/62215). When available, standardized definitions from the Brighton Collaboration were applied during reviews (*4*). Because dose number in a vaccination series is often missing or inconsistently reported in VAERS, this information was not analyzed. Vaccination errors were identified by applying a previously used error-search strategy (*5*) and included any reports with recipient age <50 years or subcutaneous route of administration. Empirical Bayesian data mining methods were used to identify RZV-adverse event pairings that were reported at least twice as frequently as were reported in all other U.S.-licensed vaccines in the VAERS database (*3*).

During the analytic period, VAERS received 4,381 RZV reports (Table 1), for a rate of 136 reports per 100,000 doses distributed; among these, 130 (3.0%) were classified as serious (four serious reports per 100,000 doses distributed). Women accounted for 2,870 (65.5%) reports. For 4,167 (95.1%) reports, RZV was the only vaccine that had been administered. Most reports were submitted by health care professionals (1,661; 37.9%) and the vaccine manufacturer (1,661; 37.9%). Pyrexia was reported most frequently (1,034; 23.6%) (Table 2). Other systemic symptoms, such as chills, headache, fatigue, and myalgia, were commonly reported, as were injection site reactions. Reported signs and symptoms were similar whether RZV was administered alone or in combination with other vaccines. Median interval from receipt of RZV to onset of signs or symptoms was 1 day (i.e., the day after vaccination). Persons aged 50–69 years reported a high proportion of systemic signs and symptoms, such as pyrexia (29.1%), chills (24.6%), and headache (21.3%), whereas persons aged ≥70 years reported a high frequency of local symptoms, such as injection site erythema (22.5%) and pain (21.5%).

**TABLE 1:** Characteristics of recombinant zoster vaccine (RZV) reports submitted to VAERS — United States, October 2017–June 2018

Report characteristic	No. (%)
**Total reports**	**4,381 (100)**
**Sex**	
Women	2,870 (65.5)
Men	1,265 (28.9)
Not reported or unknown	246 (5.6)
Seriousness*	
Nonserious	4,251 (97.0)
Serious^†^	130 (3.0)
**Type of reporter**	
Health care professional	1,661 (37.9)
Manufacturer	1,661 (37.9)
Patient	801 (18.3)
Other	236 (5.4)
Parent/Guardian/Caretaker	22 (0.5)
**Age group (yrs)**	
<50^§^	27 (0.6)
50–59	956 (21.8)
60–69	1,467 (33.5)
70–79	988 (22.6)
≥80	251 (5.7)
Not reported or unknown	692 (15.8)
**RZV given alone^¶^**	4,167 (95.1)

**Abbreviation:** VAERS = Vaccine Adverse Event Reporting System.

* Includes hospitalization, prolongation of existing hospitalization, life-threatening illness, permanent disability, congenital anomaly or birth defect, and death, as defined in Code of Federal Regulations Title 21 Section 600.80.

^†^ Includes eight reports of death, seven of which were confirmed using autopsy reports, death certificates, or medical records; one was an unconfirmed hearsay (i.e., secondhand) report.

^§^ RZV is not licensed for use in this age group.

^¶^ When RZV was given concomitantly with other vaccines, the most common vaccines included 23-valent pneumococcal polysaccharide (86); tetanus, diphtheria, acellular pertussis (Tdap), tetanus, diphtheria (Td), or tetanus toxoid (TT) (57 tetanus toxoid–containing vaccines); 13-valent pneumococcal conjugate (43); influenza (19); hepatitis A (16); and combination hepatitis A and B (seven) vaccines.

**TABLE 2:** Most commonly reported symptoms* after receipt of recombinant zoster vaccine (RZV) in reports submitted to VAERS (N = 4,381)^†^ — United States, October 2017–June 2018

Sign/Symptom	Total RZV reports, no. (%)	RZV given in combination with other vaccines, no. (%)
Pyrexia	1,034 (23.6)	57 (26.6)
Injection site pain	985 (22.5)	49 (22.9)
Injection site erythema	880 (20.1)	50 (23.4)
Pain	853 (19.5)	45 (21.0)
Chills	847 (19.3)	32 (15.0)
Headache	730 (16.7)	30 (14.0)
Fatigue	703 (16.0)	23 (10.7)
Pain in extremity	691 (15.8)	37 (17.3)
Injection site swelling	588 (13.4)	29 (13.6)
Myalgia	530 (12.1)	19 (8.9)

**Abbreviation:** VAERS = Vaccine Adverse Event Reporting System.

* According to Medical Dictionary for Regulatory Activities Preferred Terms, a single report may be assigned more than one Preferred Term (i.e., terms are not mutually exclusive).

^†^ Includes reports for RZV given alone (95.1%) and concomitantly with other vaccines.

Seven confirmed deaths after receipt of RZV were reported. According to autopsy reports, death certificates, or medical records, the median decedent age was 65 years (range = 61–86 years), and the interval from vaccination to death ranged from 6 hours to 6 weeks. The cause of death in four persons was cardiovascular disease, three of whom had multiple cardiac risk factors. Two persons, both of whom were immunosuppressed, died of septic shock. One death occurred in a woman (aged 86 years) who died subsequent to a fall.

The most commonly reported prespecified outcomes (Supplementary Table 2, https://stacks.cdc.gov/view/cdc/62215) were herpes zoster (196; 4.5%; 6.1 reports per 100,000 RZV doses distributed; 14 reports specified previous herpes zoster) and postherpetic neuralgia (49; 1.1%; 1.5 reports per 100,000 RZV doses distributed; six reports specified previous postherpetic neuralgia). The remaining prespecified outcomes each accounted for <0.5% of total reports.

Overall, 230 reports described vaccination errors; some reports described more than one error in the same report (Table 3). Most vaccination errors (143; 62.2%) were errors of administration, and among these, the most frequent error was incorrect route of administration (108; 75.5% of administration errors), with RZV given subcutaneously rather than intramuscularly. RZV is supplied with two vials that must be combined before administration. One vial contains the lyophilized antigen, and the other contains the AS01_B_ adjuvant suspension component (liquid) that is mixed with the contents of the first vial. Among 19 reports documenting product preparation errors, eight included administration of only the AS01_B_ adjuvant; 11 reported mixing RZV lyophilized antigen with the wrong diluent, including sterile water (six), ZVL diluent (four), and an unspecified incorrect diluent (one). Twenty-six reports described administration of RZV to patients aged <50 years; 15 of these reports were not coded as errors but were identified through the patient age field on the VAERS form, and therefore could represent clinical decisions to use the vaccine off-label rather than a practice error. Among 24 reports of administration of the “incorrect dose,” 12 reported an “incomplete course of vaccination,” including six cases in which health care providers advised patients who experienced common and expected adverse events (e.g., injection site reactions, arm swelling, fever, and fatigue) after the first dose of RZV to forego the second dose. Although coded as errors, these reports could represent clinical decisions by health care providers to not vaccinate, despite lack of a clear precaution or contraindication. No RZV-adverse event pairings met the statistical threshold for an empirical Bayesian data mining finding of a potential safety signal.

**TABLE 3:** Vaccination error reports (N = 230) submitted to VAERS involving recombinant zoster vaccine (RZV) — United States, October 2017–June 2018

Vaccination error group*/most common MedDRA Preferred Terms	No. (%) of reports
**Administration errors**	**143 (62.2)**
Incorrect route^†^	108
Incorrect site	26
Other^§^	9
**Inappropriate schedule**	**30 (13.0)**
Vaccine administered at inappropriate age^¶^	26
Inappropriate schedule of vaccine administration (<2 months between doses)	4
**Incorrect dose**	**24 (10.4)**
Incomplete course of vaccination	12
Incorrect dose administered**	12
**Product quality**	**23 (10.0)**
Product quality issue^††^	21
Product storage error	2
**Prescribing and dispensing**	**19 (8.3)**
Product preparation error (only adjuvant given)	8
Product preparation error (wrong diluent used)	11
**Wrong vaccine**	**4 (1.7)**
**Equipment**	**4 (1.7)**
**Product labeling and packaging**	**1 (0.4)**
**Total errors^§§^**	**248**

**Abbreviations:** MedDRA = Medical Dictionary for Regulatory Activities; VAERS = Vaccine Adverse Event Reporting System.

* Vaccination error groups contain multiple MedDRA Preferred Terms. Some reports include errors belonging to multiple error groups.

^†^ Thirty-eight of 108 reports were not coded with a MedDRA Preferred Term for an incorrect route error, but subcutaneous route was selected on the VAERS form field for route of administration.

^§^ Includes wrong technique (five) and accidental exposure to product involving vaccine splashing on the health care provider or patient skin or eyes during product preparation (four).

^¶^ Fifteen of 26 reports of RZV given to patients aged <50 years were not coded with a MedDRA Preferred Term for an inappropriate age error, but age at vaccination of <50 years was documented on the VAERS form; these 15 reports could therefore represent clinical decisions to use the vaccine off-label rather than a practice error.

** Includes incorrect dose administered (eight), overdose (too much volume) (two), accidental overdose (one), underdose (too little volume caused by patient pulling away during administration) (one).

^††^ Health care provider or patient questioning of product quality was related to adverse events after administration of RZV and not based on empiric or objective evidence of actual product quality problems.

**^§§^** A single report might describe more than one error; 230 reports described 248 errors.

## Discussion

Although VAERS data are subject to the limitations inherent in passive surveillance, the initial safety data from VAERS monitoring during the first 8 months of RZV use are consistent with the safety profile observed in prelicensure clinical trials (*2*,*6*,*7*). No adverse events reported for RZV were disproportionate to adverse event reporting patterns observed for other vaccines in the VAERS database. Reports for prespecified outcomes are generally consistent with reporting patterns observed for other vaccines in VAERS and likely represent temporally associated events that are occurring as background incidence in the general population.

Passive surveillance data are not conducive to direct comparisons between vaccines, but observations of reporting patterns can reveal general similarities and differences. Injection site reactions were commonly reported for both RZV and ZVL vaccines. Herpes zoster and rash were commonly reported for ZVL, whereas systemic reactions including pyrexia and chills were commonly reported for RZV. Reporting rates for RZV were 136 per 100,000 doses distributed (all adverse event reports) and 4.0 per 100,000 (serious adverse event reports) versus 106 and 4.4, respectively, for ZVL (*8*). Because dose number in series (i.e., first or second) is not consistently reported in VAERS, the number of reports representing a person’s first or second exposure to RZV is unknown. Of note, errors involving subcutaneous administration of RZV (the vaccine is licensed for intramuscular injection) could reflect confusion with administration procedures for ZVL, which is administered subcutaneously.

Several reports suggested that health care providers made clinical decisions to not administer the second dose of RZV after observing local or systemic reactions in patients. In clinical trials, approximately 17% of RZV recipients experienced grade 3 reactions (*2*,*6*,*7*); these episodes were self-limited and resolved in a few days. Providers should expect such reactions in many of their patients and counsel them accordingly. The effectiveness of a single dose of RZV has not been studied.

CDC and FDA will continue to closely monitor the safety of RZV. Whereas the initial safety data for RZV are reassuring, the vaccine is still in the early uptake period. Understanding of the safety of RZV will advance as use increases and additional data become available from VAERS and from near real-time sequential monitoring in CDC’s Vaccine Safety Datalink (*9*).

**Summary**

**What is already known about this topic?**

Recombinant zoster vaccine (RZV), a highly efficacious shingles vaccine licensed in October 2017, is recommended for adults aged ≥50 years. In clinical trials, local and systemic vaccine reactions were common.

**What is added by this report?**

Early RZV safety monitoring findings are consistent with prelicensure clinical trial data. Serious adverse events were rare, and no unexpected patterns were detected.

**What are the implications for public health practice?**

Health care providers and patients can be reassured by RZV’s initial postlicensure safety data. Counseling patients to expect self-limited adverse reactions such as pain, swelling and redness at the injection site, fever, chills, and body aches might ease concerns and encourage completion of the 2-dose RZV series.

## Footnotes

* A distinct descriptor (e.g., for a symptom, sign, or disease diagnosis). https://www.meddra.org/how-to-use/basics/hierarchy.

† https://www.accessdata.fda.gov/scripts/cdrh/cfdocs/cfcfr/CFRSearch.cfm?fr=600.80.
